# Efficacy and Safety of Ketamine-Dexmedetomidine Versus Ketamine-Propofol Combination for Periprocedural Sedation: A Systematic Review and Meta-analysis

**DOI:** 10.1007/s11916-023-01208-0

**Published:** 2024-01-12

**Authors:** Ahmed Saad Elsaeidy, Aya Hisham Moussa Ahmad, Neveen A. Kohaf, Aya Aboutaleb, Danisha Kumar, Khaled Saad Elsaeidy, Ola saeed Mohamed, Alan D. Kaye, Islam Mohammad Shehata

**Affiliations:** 1 Faculty of Medicine, Benha University, Benha, Egypt; 2 Anesthesia Department, Faculty of Medicine, Ain Shams University, Cairo, Egypt; 3 Clinical Pharmacy, Faculty of Pharmacy (Girls), Al-Azhar University, Cairo, Egypt; 4 Faculty of Medicine, Zagazig University, Zagazig, Egypt; 5 Dow Medical College, Dow University of Health Sciences, Karachi, Pakistan; 6 Faculty of Medicine, Mansoura University, Mansoura, Egypt; 7 Critical Care Medicine, Menofia University, Shibin El Kom, Menofia, Egypt; 8 Pharmacology, Toxicology, and Neurosciences, LSU School of Medicine, 1501 Kings Hwy, Shreveport, LA 71103 USA; 9 Anesthesiology and Pharmacology, LSU School of Medicine, New Orleans, LA USA; 10 Anesthesiology and Pharmacology, Tulane School of Medicine, New Orleans, LA USA

**Keywords:** Ketamine, Dexmedetomidine, Propofol, Pain, Ketadex, Ketofol, Sedation

## Abstract

**Purpose of Review:**

The combination of ketamine with propofol and dexmedetomidine has gained popularity for sedation and general anesthesia in different populations. In our meta-nalysis, we helped the anesthesiologists to know the efficiency and the efficacy of both combinations in adult and pediatric patients.

**Methods:**

We searched PubMed, CENTRAL, Web of Science, and Scopus from inception to August 1, 2023. Our outcome parameters for efficacy were recovery time, pain score, and physician satisfaction while for safety were the related cardiorespiratory, neurological, and gastrointestinal adverse events.

**Recent Findings:**

Twenty-two trials were included with a total of 1429 patients. We found a significantly longer recovery time in the ketadex group of 7.59 min (95% CI, 4.92, 10.26; *I*^2^ = 94%) and a significantly less pain score of − 0.72 (95% CI, − 1.10, − 0.34; *I*^2^ = 0%). Adults had a significantly better physician satisfaction score with the ketofol group, odds ratio of 0.29 (95% CI, 0.12, 0.71; *I*^2^ = 0%). Recovery agitations were higher in the ketofol group with an odds ratio of 0.48 (95% CI, 0.24, 0.98; *I*^2^ = 36%). Furthermore, we found a significant difference between the combinations with a higher incidence in the ketadex group with pooled odds ratio of 1.75 (95% CI, 1.06, 2.88; *I*^2^ = 15%).

**Summary:**

Ketadex was associated with lower pain scores, hypoxic events and airway obstruction, and emergence agitation. At the same time, ketofol had much more clinician satisfaction which might be attributed to the shorter recovery time and lower incidence of nausea and vomiting. Therefore, we suppose that ketadex is the better combination in periprocedural sedation for both adult and pediatric patients who are not at greater risk for postoperative nausea and vomiting.

**Supplementary Information:**

The online version contains supplementary material available at 10.1007/s11916-023-01208-0.

## Introduction

With the current shift towards day case surgeries, office-based procedures, and minimally invasive diagnostic procedures and interventions, there is an increasing demand for safe and effective sedation and/or anesthesia regimen that is short-acting and provides a favorable recovery profile with minimal side effects [1].

Several agents have been tested as sole or combined [2–5]. Recently, the combination of ketamine with propofol (ketofol) or dexmedetomidine (ketadex) has been used for sedation and general anesthesia induction and maintenance for short procedures in different populations. These combinations minimize the side effects of each individual drug, while benefiting from combined desirable effects.

Despite being short-acting with a favorable recovery profile and anti-emetic properties, propofol can still cause hypotension and dose-dependent respiratory depression [2]. Besides sedative effects, dexmedetomidine possesses excellent analgesic properties but can induce hypotension and bradycardia [6]. Owing to sympathomimetic properties, ketamine increases blood pressure and heart rate and preserves respiratory activity [7]. Since ketamine has opposing cardiovascular and respiratory influences on both dexmedetomidine and propofol, the ketamine-dexmedetomidine combination (ketadex) and ketamine-propofol combination (ketofol) may be of benefit in providing satisfactory sedation and anesthesia induction and maintenance, while maintaining hemodynamic stability and reducing potential side effects of each drug.

Several studies have been conducted comparing these two combinations in the pediatric population, and recently, they started gaining popularity among the adult population, too [8, 9]. Despite the extensive research that has been done comparing them regarding sedation/anesthetic qualities and potential side effects, only one meta-analysis has been conducted in the pediatric population [10•], while none was conducted on adults.

Therefore, this meta-analysis aimed to compare the safety and efficacy of ketadex and ketofol used for procedural sedation and anesthesia for short procedures in both adult and pediatric patients.

## Methods and Materials

The present investigation was conducted in accordance with Preferred Reporting Items for Systematic Reviews and Meta-Analyses (PRISMA) recommendations [11] and the Cochrane Handbook for Systematic Reviews and meta-analysis [12]. We registered the review on Prospero (CRD42023463191).

### Search Strategy

The present investigation searched PubMed, Cochrane CENTRAL, Web of Science, and Scopus from inception to August 1, 2023, using MeSH terms and keywords for Propofol, Dexmedetomidine, Ketamine, Ketadex, and Ketofol. The supplementary table S1 overviews the search strategy we used.

### Eligibility Criteria

The present investigation included all clinical trials to compare the efficacy and safety of ketadex versus ketofol without any limitations about language, publication time, gender, age, or dosage. Any patient requiring sedation or anesthesia for any diagnostic or therapeutic procedure was included.

We excluded studies that used dexmedetomidine, propofol, or ketamine only in the intervention group or the control group. Also, we excluded all animal studies, observational studies, 2ry research (reviews, meta-analyses), letters, and conference abstracts.

### Data Extraction

Independent authors (K.S.E, A.A, D.K, O.S.M, and N.A.K) extracted the data of all included studies, and then, all extracted data were reviewed by (A.S.E and A.A). The following data were extracted: year of publication, country, number of patients in each group, age, gender, weight, the procedure, and its duration.

The same authors independently extracted data for outcomes, including recovery time, pain score, clinician satisfaction, and side effects, including the rates of hallucination, tachycardia, bradycardia, hypertension, hypotension, bradypnea, agitation, airway obstruction, salivation, nausea and/or vomiting, and hypoxia.

### Risk of Bias Assessment

Independent authors (A.H.M.A, O.S.M, and N.A.K) used the Cochrane risk of bias (ROB-2) assessment tool outlined in Chapter 8.5 of the Cochrane Handbook [11, 12]. This instrument can identify selection, performance, detection, attrition, and reporting biases. We categorized each domain’s contained articles as having low, some concerns, or high bias levels. Then, (D.K, K.S.E, and A.S.E) resolved any conflict in this task.

### Data Analysis

Continuous and dichotomous data were extracted and pooled as mean difference (MD) and odds ratio (OR) with a 95% confidence interval (CI). We used the inverse-variance (IV) method to pool effect estimates using a random effect model. According to the Cochrane Handbook (Chapter 10), we employed the chi-square and *I*^2^ tests, where the chi-square test assesses the presence of heterogeneity, and the *I*^2^ test assesses its degree. We interpreted the *I*^2^ test as follows: not significant for 0–40%, moderate heterogeneity for 30–60%, substantial heterogeneity for 50–90%, and considerable heterogeneity for 75–100% [12].

## Results

### Study Characteristics

**Fig. 1:**
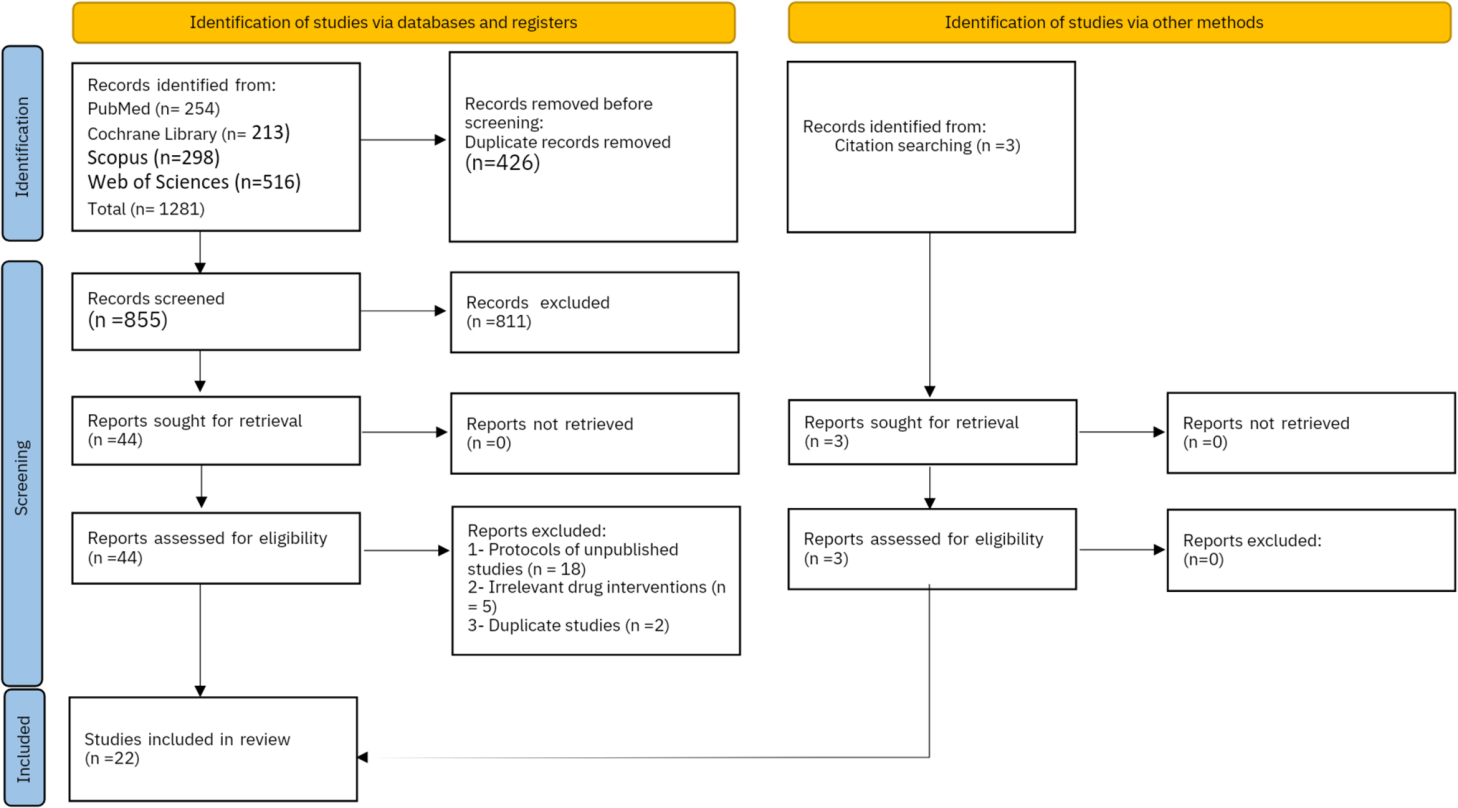
PRISMA flow diagram

Twenty-two studies were included in our review out of 1281 studies (Fig. 1). With a total of 1429 patients, 703 patients in the ketadex group and 726 patients in the ketofol group. Patients’ age ranged between 2.4 ± 1.2 and 54.9 ± 5.3 years old. Patients in 13 included studies were ≥ 18 years old. Patients were scheduled for procedures such as gastrointestinal endoscopy, cardiac catheterization, elective daycare surgeries, and some other painful procedures in the emergency department. More details about the characteristics and summary of the included studies are shown in Table 1.

**Table 1:** Summary and baseline characteristics of the included studies

Study ID	Country	Sample Siz, KD/KP	Age (year) M (SD), KD/KP	Gender, male, KD/KP	Weight (Kg) M (SD), KD/KF	Procedure	Procedure duration (Min), M (SD), KD/KP
Shetabi et al. (2023)	Iran	26/26	7.19 (3.8)/6.9 (3.9)	15/18	29.2 (16)/25.7 (16.8)	Upper gastrointestinal endoscopy	20.7 (12.4)/21.6 (10.3)
Bachula et al. (2023)	India	30/30	5.5 (2.3)/5.9 (2.8)	NA	14.5 (5.3)/15.9 (6.3)	Elective daycare surgeries: circumcision, cystoscopy, herniotomy, urethral calibration, I&D, and suturing	30 (NA)/30 (NA)
Raj et al. (2022)	India	33/34	27.09 (4.6)/27 (3.6)	NA	NA	Postoperative obstetric patients	NA
Makwana et al. (2022)	India	38/37	37.11(12.6)/42.9(14.6)	NA	59.9 (7.3)/60.3 (9.8)	Upper limb surgeries	NA
Singh et al. (2022)	India	42/42	43.83 (15.4)/49.7 (16.9)	22/22	NA	Endoscopic retrograde cholangiopancreatography	46.7 (19.2)/46.7 (19.2)
Yeter et al. (2022)	Turkey	30/30	40 (17)/45 (15)	15/18	74 (15)/75 (16)	Electro-convulsive therapy	0.7 (0.4)/0.4 (0.3)
Algharabawy et al. (2021)	Egypt	35/35	44.3 (6.8)/46.6 (3.8)	25/24	84.5 (4.3)/82.3 (3.9)	Upper gastrointestinal endoscopy	15.3 (3.6)/14.1 (2.9)
Azizkhani et al. (Aug. 2021)	Iran	31/31	7.3 (3.7)/9 (5)	18/20	25.9 (11.9)/33.3 (20.1)	Painful procedures in emergency department	10.2 (3.1)/10.1 (3.4)
Azizkhani et al. (July 2021)	Iran	31/31	39 (18)/42 (17)	24/25	71 (11)/75 (26)	Painful procedures in emergency department	12 (3)/12 (3)
Joshi et al. (2020)	India	15/15	NA	NA	NA	Dental treatment	NA
Saini et al. (2020)	India	50/50	43.7 (9.9)/45.1 (10.7)	19/21	70.3 (6.7)/69.5 (5.8)	Laparoscopic Cholecystectomy	54.8 (6.7)/52.9 (7.9)
Amer et al. (2020)	Egypt	60/60	3.5 (1.6)/4.3 (1.7)	30/24	15 (4)/17.3 (5.6)	Upper gastrointestinal endoscopy	5.7 (2.2)/5.6 (1.9)
El Sharkawy et al. (2019)	Egypt	30/30	43.6 (11.6)/40.8 (13.5)	17/14	NA	Elective surgery under General Anesthesia	NA
Sree et al. (2019)	India	31/29	3.3 (3.8)/4.2 (4.6)	12/17		Cardiac catheterization	94 (45.1)/92 (47)
Mogahd et al. (2017)	Egypt	35/35	53.5 (4.9)/54.9 (5.3)	18/20	79.7 (6.4)/81.4 (6.9)	Coronary artery bypass graft surgery	NA
Canpolat et al. (2017)	Turkey	30/30	5.3 (1.7)/5.4 (1.4)	18/17	20.9 (7.1)/19.7 (4.5)	Dental treatment	7.5 (3)/7.8 (3.8)
Joshi et al. (2017)	India	30/30	4.84 (2.6)/5.1 (2.2)	NA	15.5 (6.3)/16.6 (5.4)	Cardiac minor procedures and catheterization	44 (10.8)/39.2 (11.7)
Simsek et al. (2016)	Turkey	20/20	4.37 (2.9)/5.3 (2.9)	7/11	17.2 (14.7)/20.1 (10.2)	Cardiac catheterization	44.4 (25.4)/53.9 (24.1)
Ali et al. (2014)	India	29/30	4.3 (3)/4.5 (3.3)	14/12	13.1 (5.5)/15.4 (9.7)	Cardiac catheterization	NA
Shaaban et al. (2014)	Egypt	20/20	6–12 years, range	15	NA	Invasive oncology procedures	NA
Canpolat et al. (2012)	Turkey	30/30	2.4 (1.5)/2.4 (1.2)	21/19	13.6 (3.7)/13.3 (3.3)	Burn wound dressing changes	11 (4.8)/10.3 (5.5)
Tosun et al. (2006)	Turkey	22/22	7.08 (3.9)/6.3 (4.7)	10/10	24.6 (18.6)/19.6 (13.5)	Cardiac catheterization	42.8 (20.9)/52.1 (20.9)

Data presents as mean (standard deviation) of ketadex group/ketofol group

*NA* non-available data

### Quality Assessment

**Fig. 2:**
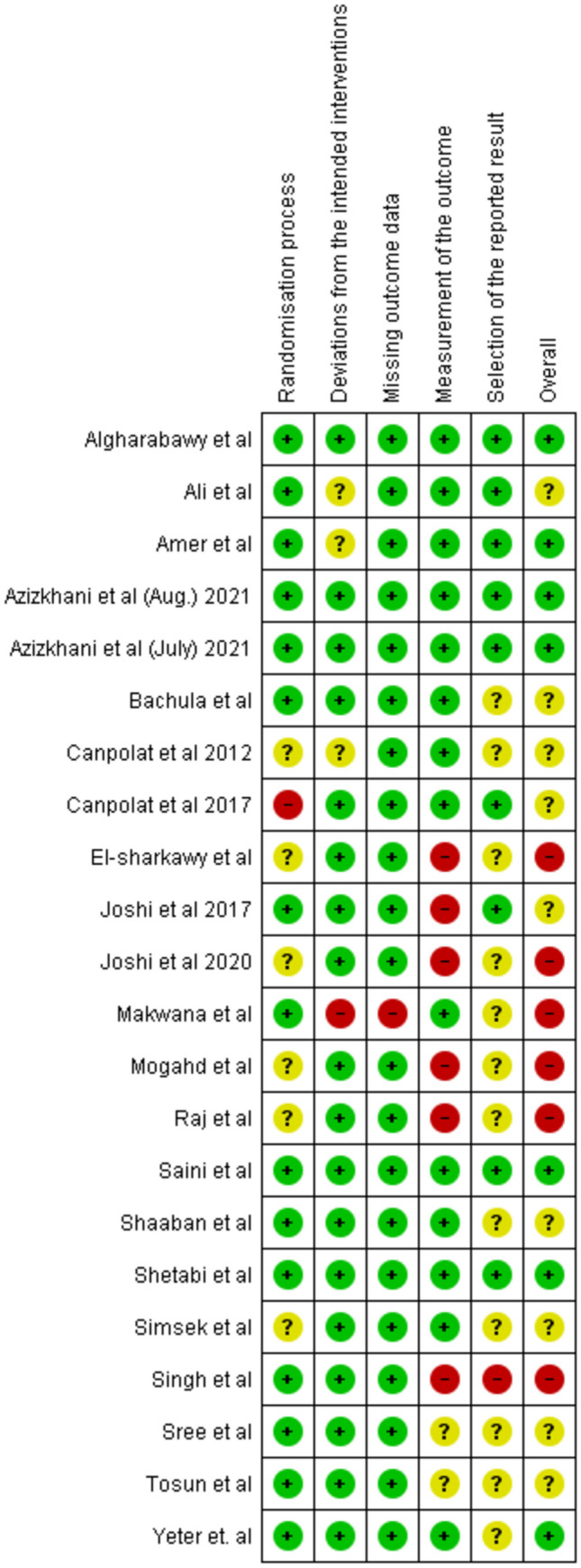
Summary of Risk of Bias in the included studies

Risk of bias assessment of the 22 included studies indicated a high risk of bias in six studies [8, 9, 13–16], an unclear risk of bias in nine studies [17–25], and a low risk of bias in seven studies [26–32], as shown in (Fig. 2).

### Efficacy Outcomes

#### Recovery Time

**Fig. 3:**
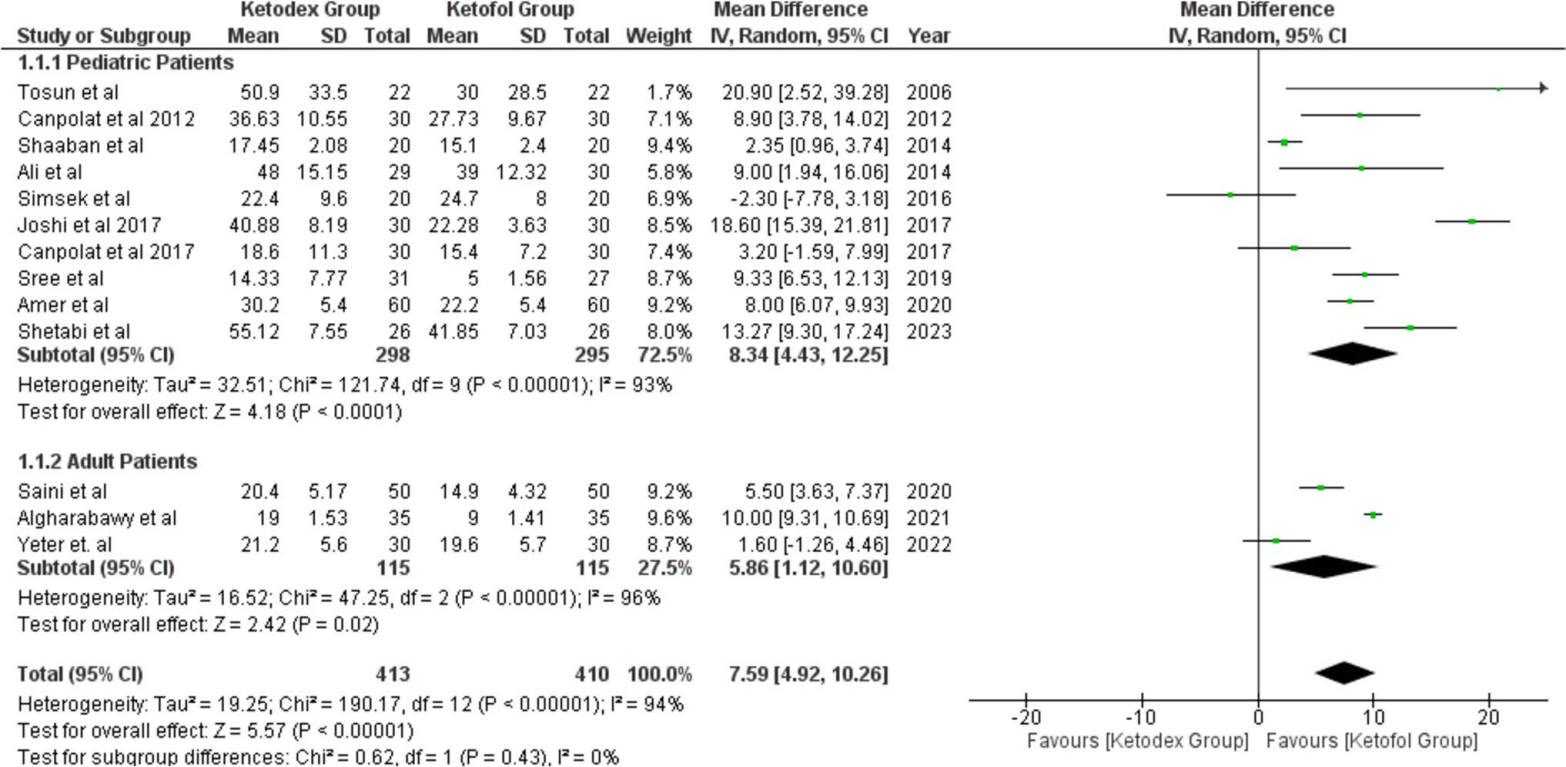
Forest plot of Recovery time outcome with age subgroups

Thirteen studies reported the recovery time by minutes after the surgery in 823 patients (413 ketadex vs 410 ketofol). The pooled mean difference showed a significant difference between the two groups of 7.59 min (95% CI, 4.92, 10.26; *I*^2^ = 94%) indicating longer recovery time in the ketadex group. Additionally, subgroup analysis showed a significant difference between the two groups with the ketadex group having a longer recovery time in pediatrics and adults at 8.34 min (95% CI, 4.43, 12.25; *I*^2^ = 93%) and 5.86 min (95% CI, 1.12, 10.60; *I*^2^ = 96%), respectively (Fig. 3).

Furthermore, we performed subgroup analysis according to the procedure and recovery score. Seven studies used the combinations for cardiac catheterization and three studies for UGIE showed a significant mean difference that favors ketofol 10.08 min (95% CI, 2.49, 17.67; *I*^2^ = 91%) and 9.86 min (95% CI, 7.86, 11.86; *I*^2^ = 70%) (Fig. S1). Six studies used Steward Score ≥ 6 and three studies used Aldrete Score ≥ 9 showed a significant mean difference that favors ketofol (Fig. S2). All included studies showed longer recovery time with the ketadex group irrespective of the type of the procedure and recovery score, except for data from three studies (Yeter et al. 2012, Smisek 2016, and Canpolat 2017) in which the difference was not of statistical significance.

#### Pain Score

**Fig. 4:**
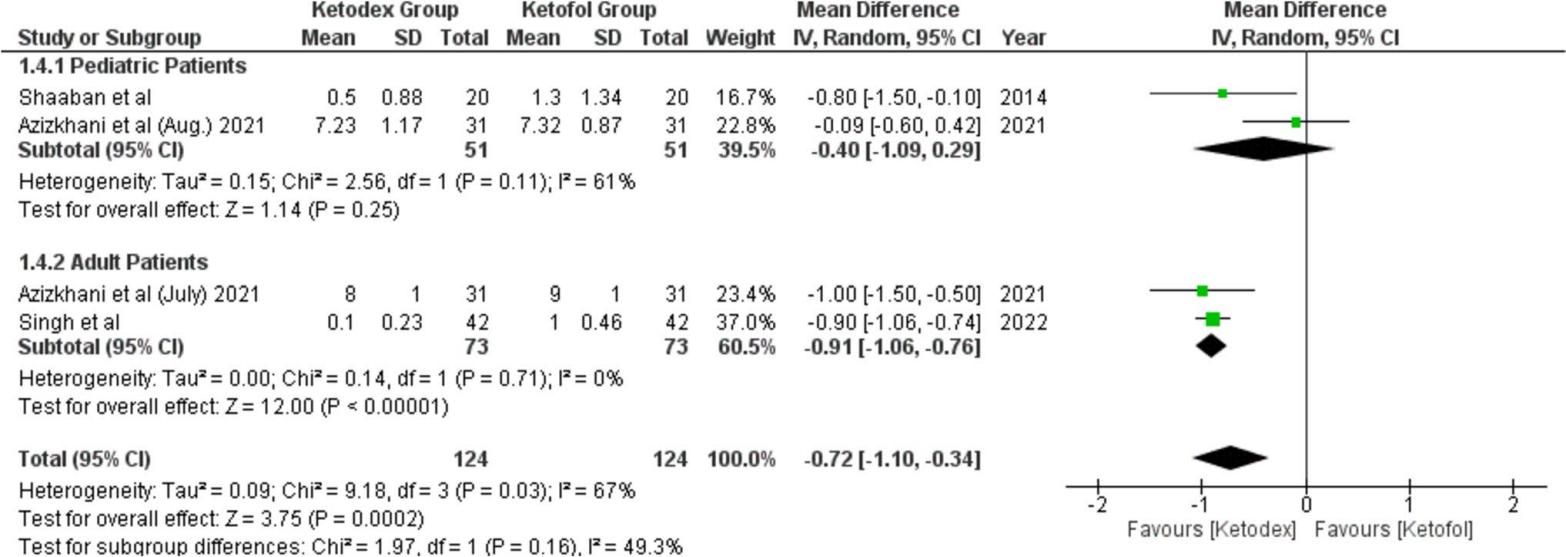
Forest plot of Pain score outcome with age subgroups

Four studies reported the pain score by the visual analogue scale (Fig. 4) and showed that 248 patients experienced less pain with the ketadex group than with the ketofol group. The pooled mean difference was statistically significant − 0.72 (95% CI, − 1.10, − 0.34; *I*^2^ = 0%). Both age groups experienced less pain in the ketadex group, but the mean difference was significantly lower in adults and the insignificant difference in pediatrics − 0.91 (95% CI, − 1.06, − 0.76; *I*^2^ = 0%) and − 0.40 (95% CI, − 1.09, 0.29; *I*^2^ = 61%), respectively (Fig. 4). Azizkhani et al. used the combinations for painful procedures in the emergency room in two studies with no pooled difference between the combinations − 0.55 (95% CI, − 1.44, 0.34; *I*^2^ = 84%). However, Azizkhani et al. (July) 2021 found significantly less pain in the ketadex group (Fig. S3).

#### Physician Satisfaction

**Fig. 5:**
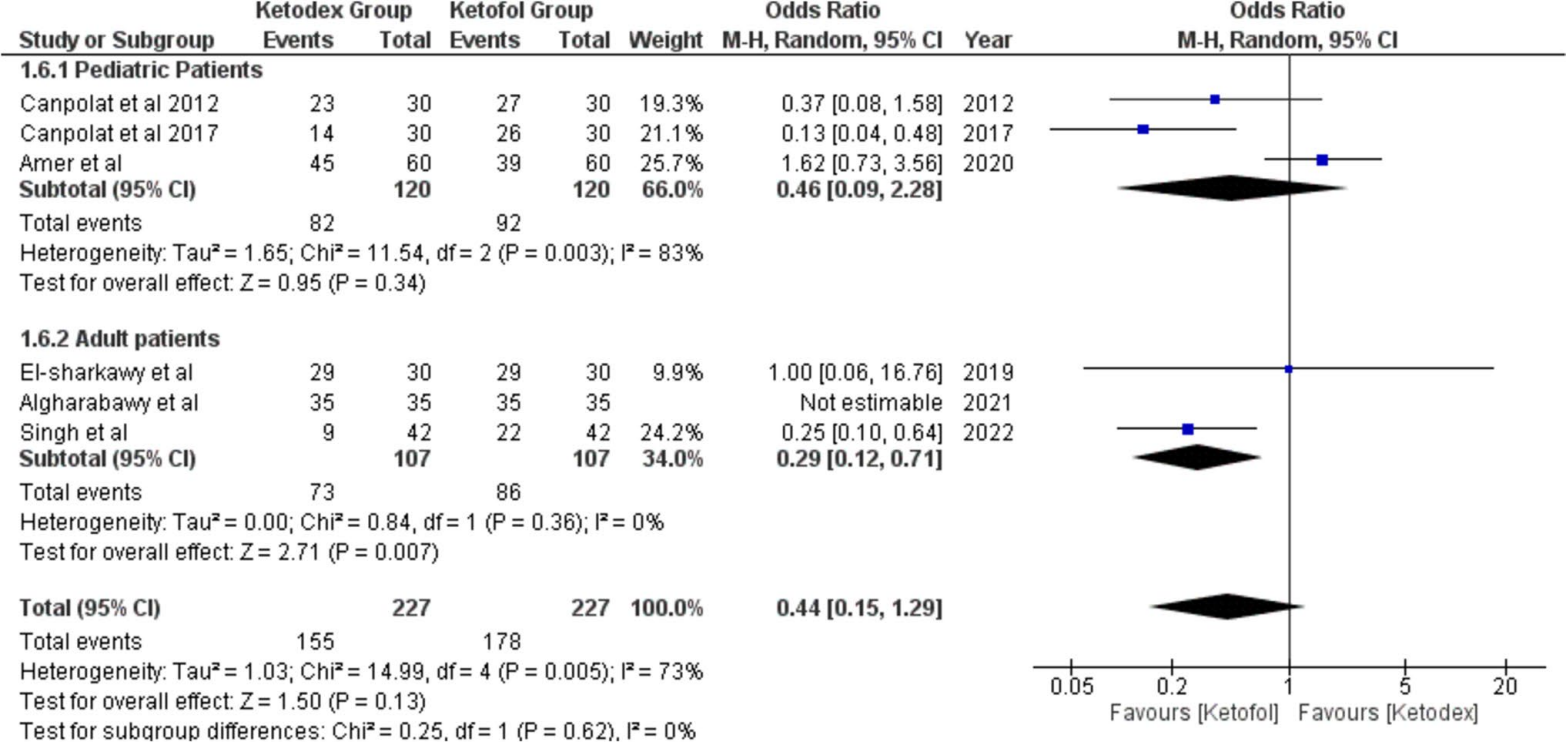
Forest plot of Physician satisfaction outcome with age subgroups

Good or Excellent physician satisfaction scores were reported in six studies. The pooled odds ratio was not statistically significant, being 0.44 (95% CI, 0.15, 1.29; *I*^2^ = 73%). Adults had a significantly better physician satisfaction score with the ketofol group, odds ratio of 0.29 (95% CI, 0.12, 0.71; *I*^2^ = 0%). But in pediatrics, the pooled odds ratio showed insignificant better physician satisfaction with ketofol in pediatrics (Fig. 5). After the removal of Amer et al. as a potential cause of heterogeneity, we found significant pooled odds ratio in total events and pediatrics 0.25 (95% CI, 0.13, 0.47; *I*^2^ = 0%) and 0.21 (95% CI, 0.08, 0.53; *I*^2^ = 2%) with no heterogeneity between the studies (Fig. S4).

### Safety Outcomes:

#### Cardiovascular Adverse Events

**Fig. 6:**
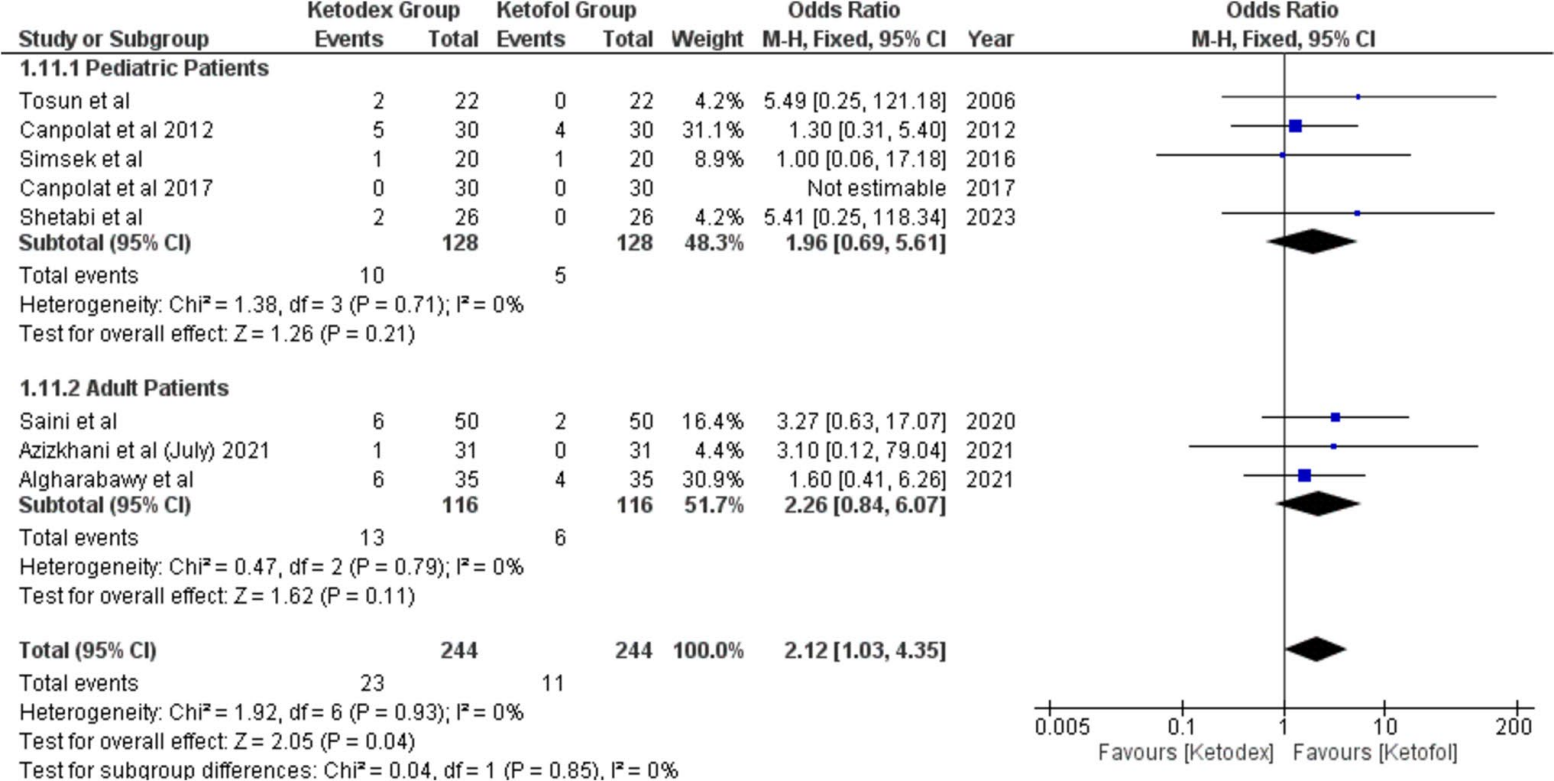
Forest plot of Bradycardia outcome with age subgroups

Bradycardia events were reported in eight studies of 488 children. The incidence of bradycardia was significantly higher in the ketadex group at 9.43% than in the ketofol group at 4.5%, odds ratio of 2.12 (95% CI, 1.03, 4.35; *I*^2^ = 0%). The difference between the two combinations was not statistically significant in both age subgroups (Fig. 6), and the procedure type (Fig. S5).

**Fig. 7:**
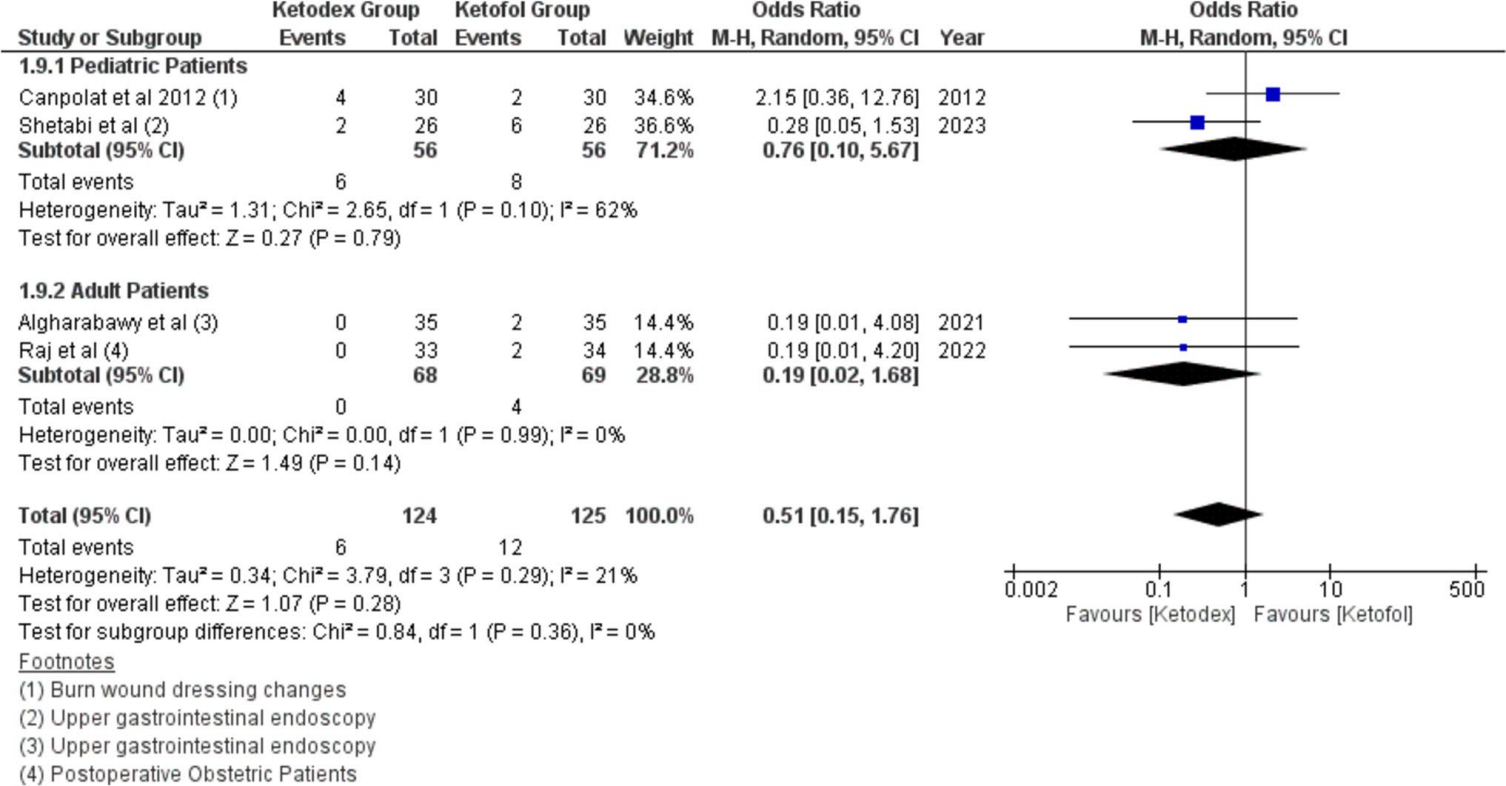
Forest plot of Tachycardia outcome with age subgroups

Tachycardia events were reported in four studies of 249 children. The incidence of Tachycardia was more in the ketofol group 9.6% compared to the ketadex group 4.8% with pooled odds ratio of 0.51 (95% CI, 0.15, 1.76; *I*^2^ = 0%), but there was no statistically significant difference between the two combinations in both adults and pediatrics (Fig. 7). The incidence of Tachycardia was insignificant more with the ketofol group after upper gastrointestinal endoscopy, odds ratio of 0.25 (95% CI, 0.06, 1.11; *I*^2^ = 0%) (Fig. S6).

**Fig. 8:**
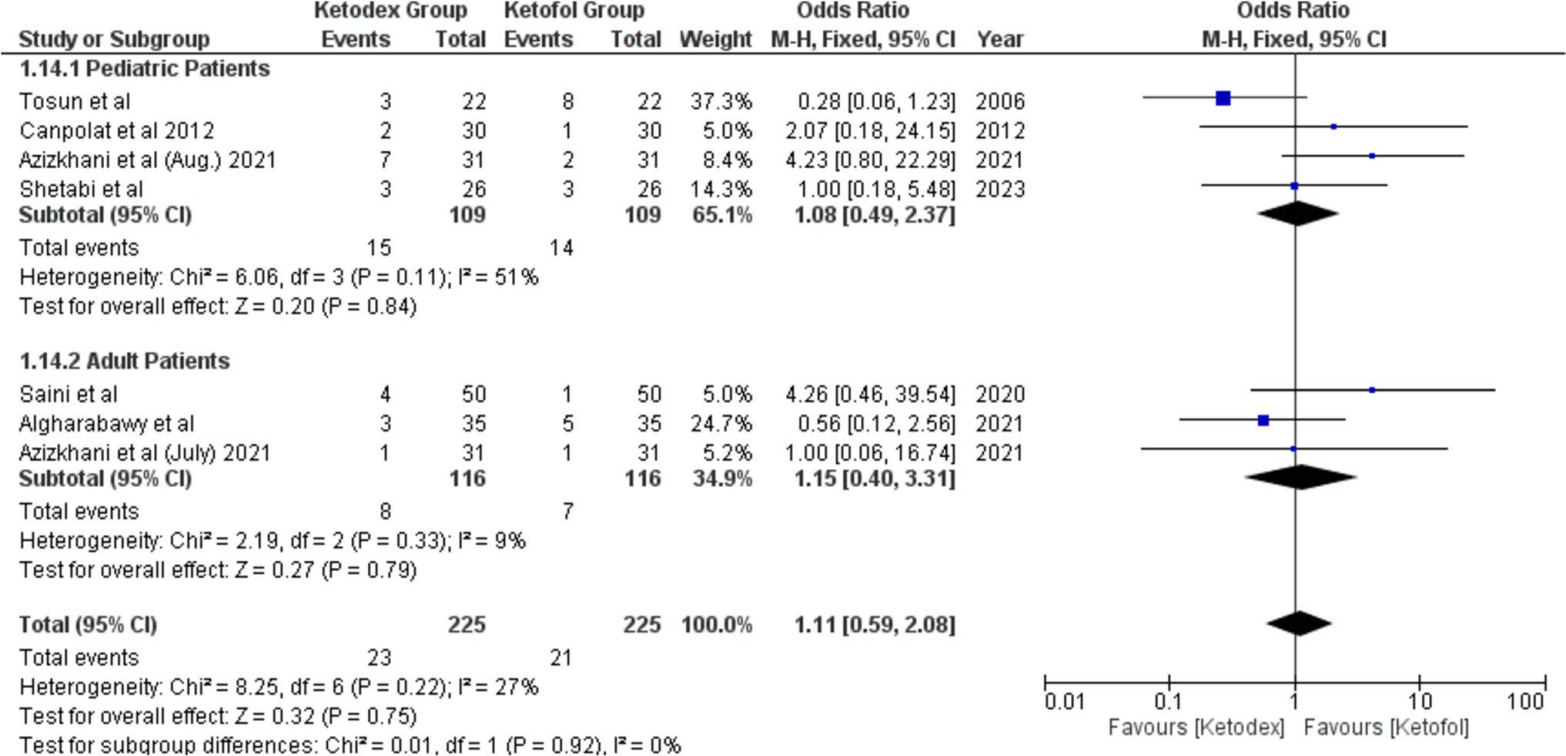
Forest plot of Hypotension outcome with age subgroups

**Fig. 9:**
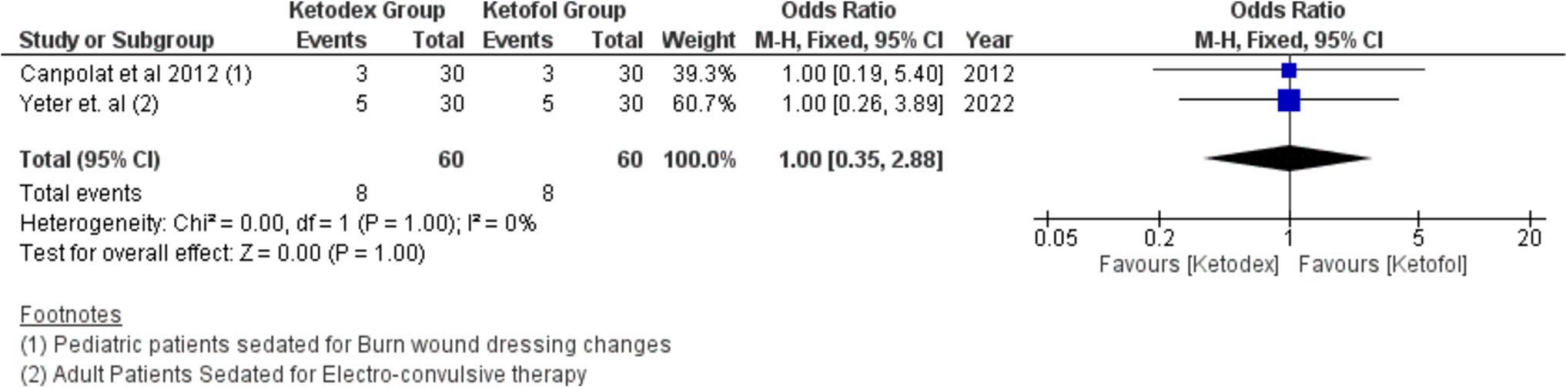
Forest plot of Hypertension outcome with age subgroups

Hypotension events were reported in 44 patients. The difference in hypotension incidence between the two combinations was not of statistical significance, odds ratio of 1.11 (95% CI, 0.59, 2.08; *I*^2^ = 27%) (Fig. 8). Also, hypertension events from two studies reported the same incidence in both combinations leading to statistically insignificant difference, odds ratio of 1.00 (95% CI, 0.35, 2.88; *I*^2^ = 0%, *P* = 1.00) (Fig. 9).

#### Respiratory Adverse Events

**Fig. 10:**
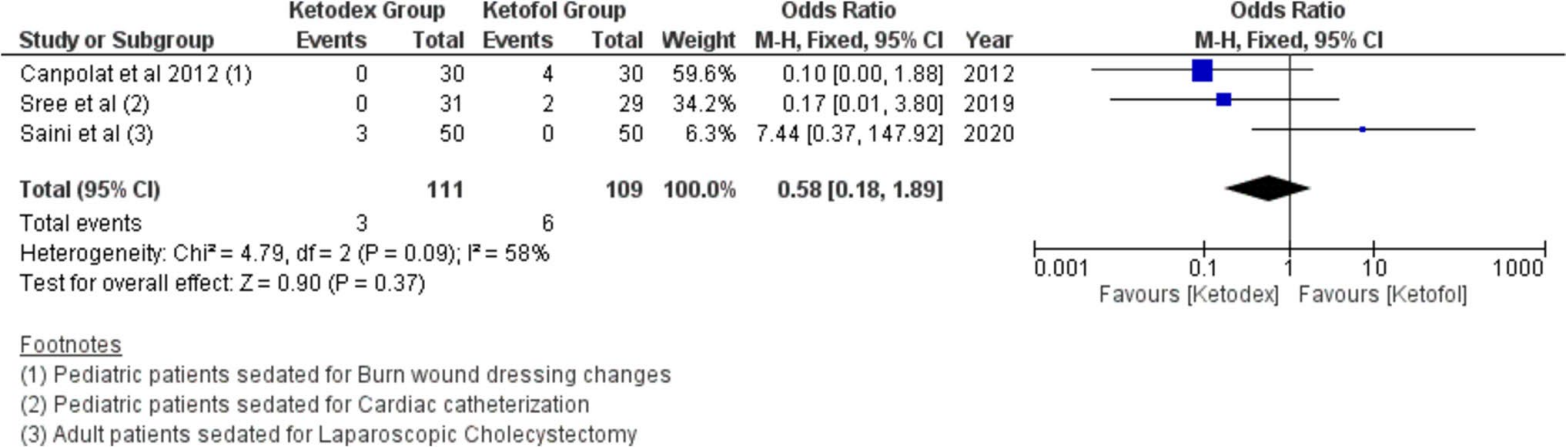
Forest plot of Bradypnea outcome with age subgroups

**Fig. 11:**
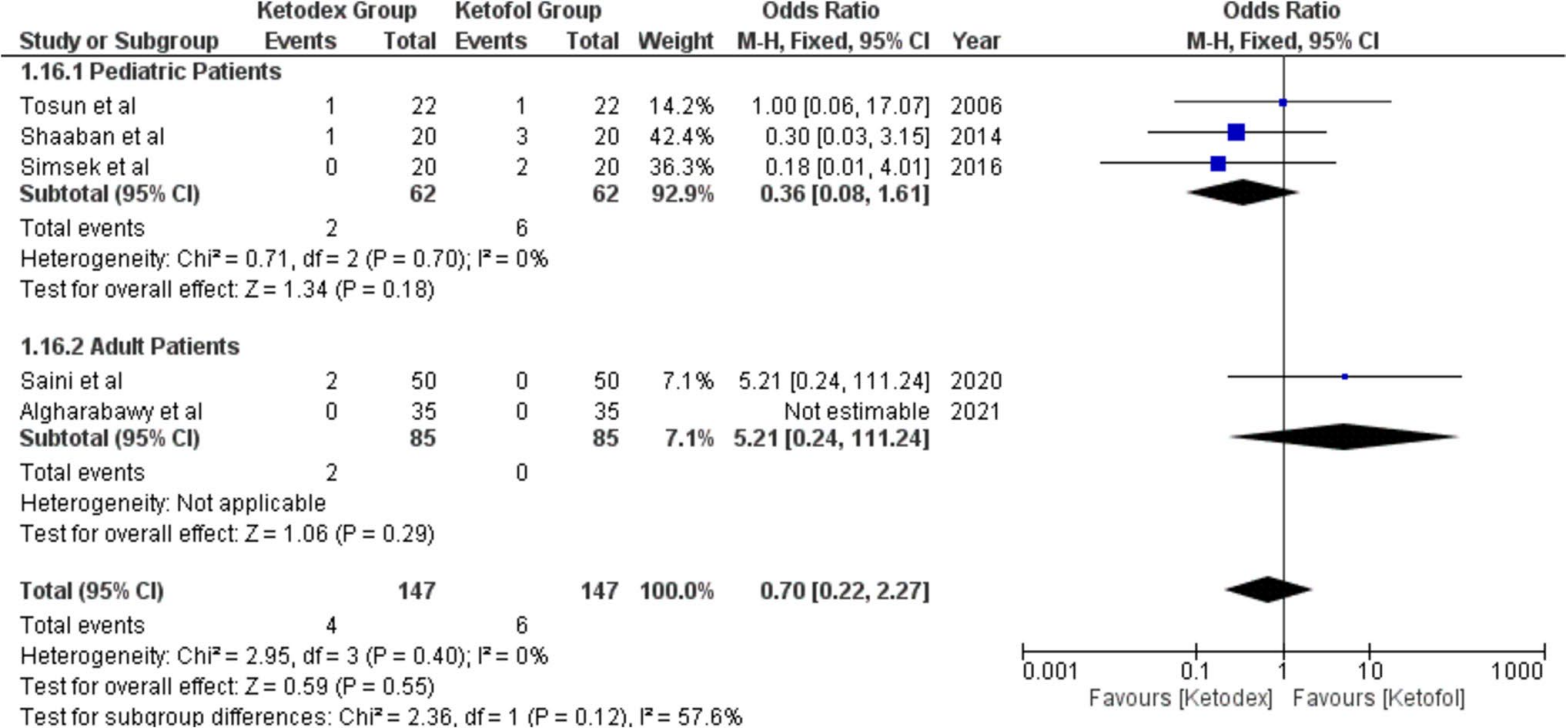
Forest plot of Airway Obstruction outcome with age subgroups

Bradypnea events were reported in nine patients of three studies. The incidence of Bradypnea in the ketadex was 2.7% while in the ketofol was 5.5%. There was no significant difference between the combinations with an odds ratio of 0.58 (95% CI, 0.18, 1.89; *I*^2^ = 58%) (Fig. 10). Additionally, airway obstruction events showed a significant difference between the combinations with an odds ratio of 0.70 (95% CI, 0.22, 2.27; *I*^2^ = 0%) (Fig. 11).

**Fig. 12:**
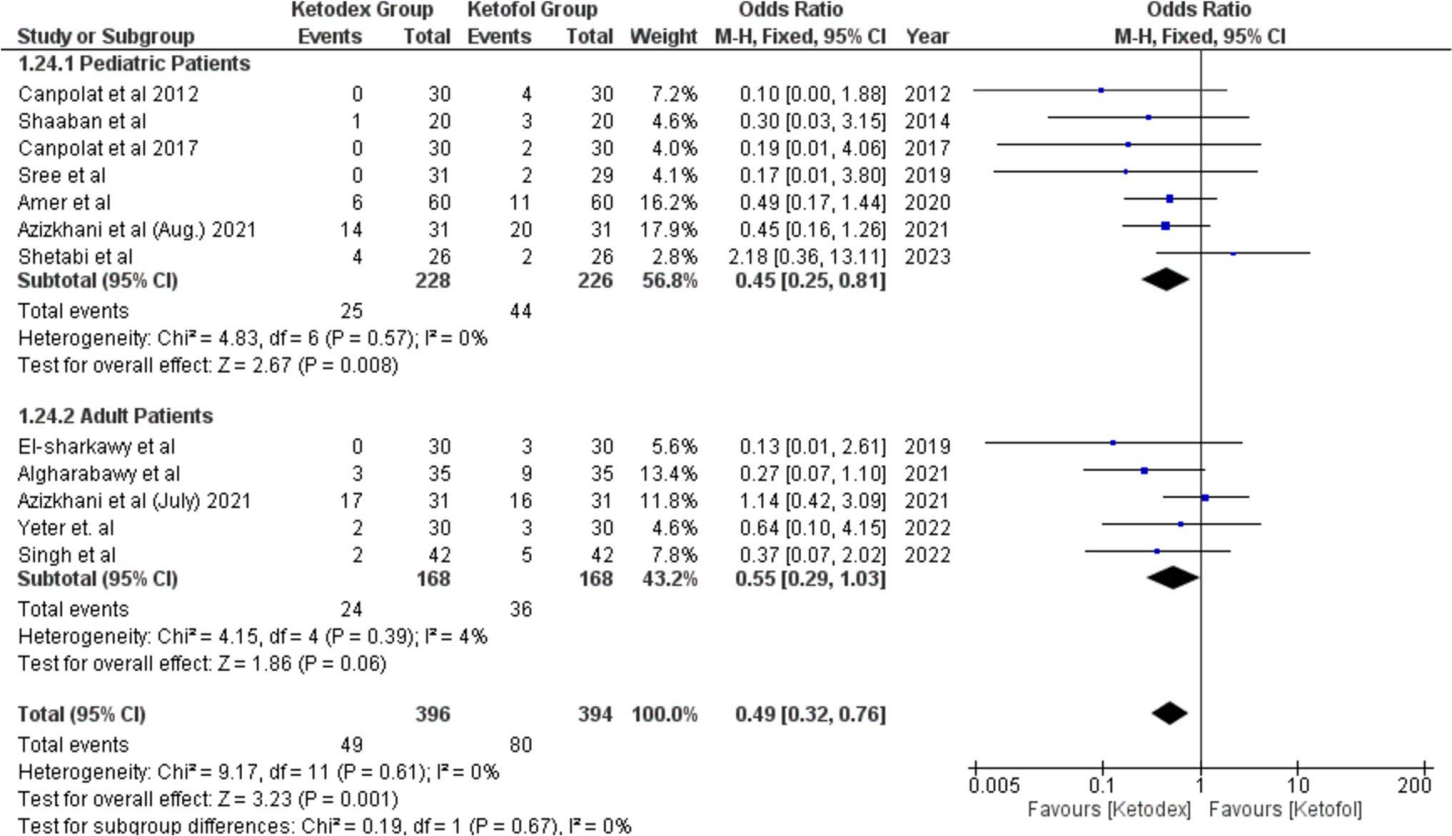
Forest plot of Hypoxia outcome with age subgroups

Hypoxia events were reported in 129 patients of 12 studies. The incidence of hypoxia was lower in the ketadex group 12.4% than in the ketofol group 20.3%; the difference between the two groups was statistically significant with an odds ratio of 0.49 (95% CI, 0.32, 0.76; *I*^2^ = 0%). The difference in the incidence of hypoxia was not statistically significant between the combinations in adult patients, odds ratio of 0.55 (95% CI, 0.29, 1.03; *I*^2^ = 4%), while the difference remains statistically significant in the pediatric patients, odds ratio of 0.45 (95% CI, 0.25, 0.81; *I*^2^ = 0%) (Fig. 12). The incidence of hypoxia remained higher in the ketofol group throughout all different types of procedures, with no statistically significant difference between the two groups in any type of procedure (Fig. S7).

#### Gastrointestinal Adverse Events

**Fig. 13:**
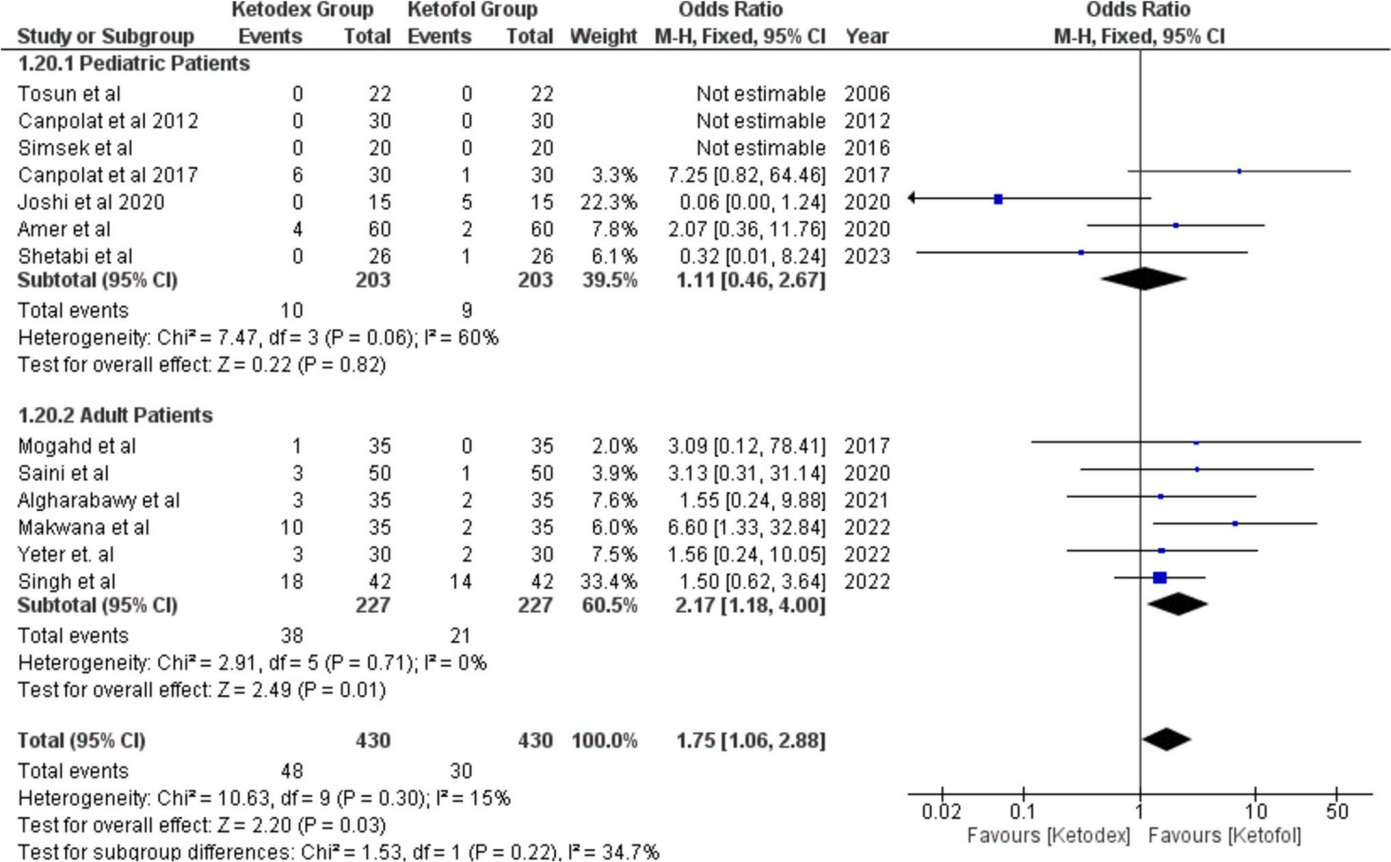
Forest plot of Post-operative Nausea and/or vomiting with age subgroups

Post-operative nausea and/or vomiting (PONV) was addressed as a single side effect in most of the studies, so we did the same in our analysis. Thirteen studies reported PONV in 78 patients. We found a statistically significant difference between the combinations with a higher incidence in the ketadex group11.2% than in the ketofol group 7% with pooled odds ratio of 1.75 (95% CI, 1.06, 2.88; *I*^2^ = 15%). Also, we found that ketadex caused statistically significant higher incidence in the adult group with pooled odds ratio of 2.17 (95% CI, 1.18, 4.00; *I*^2^ = 0%), while there was no statistically significant difference between both combinations in the pediatrics (Fig. 13).

PONV occurred more in the ketadex group regardless of the type of the procedure, except with dental treatment, which showed the same incidence in both groups (Fig. S8).

**Fig. 14:**
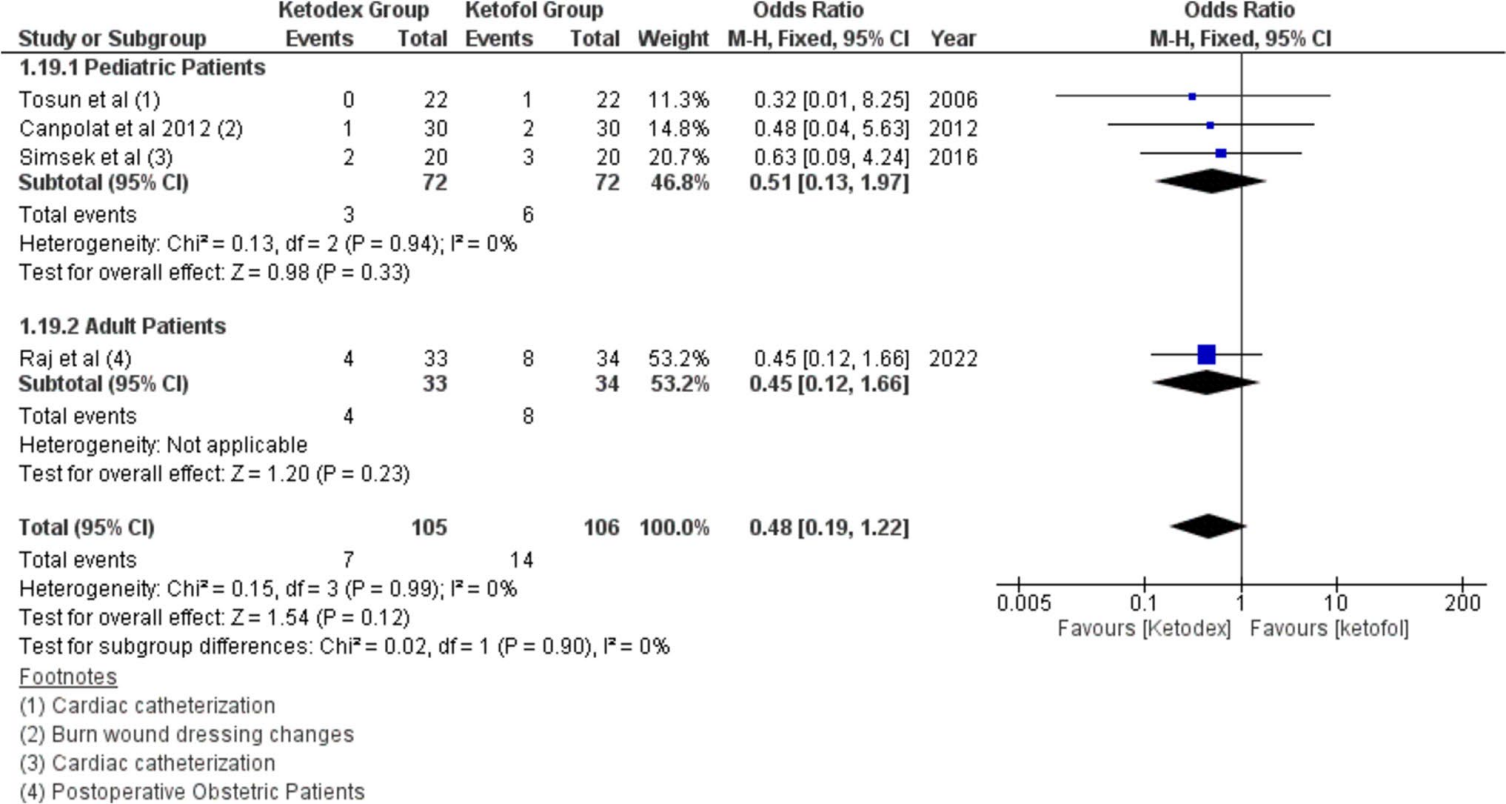
Forest plot of Salivation outcome with age subgroups

Salivation events were reported in 21 patients of four studies. The incidence of salivation was higher in the ketofol group 13.2% than in the ketadex group 6.7%. However, we found a statistically insignificant pooled odds ratio of 0.48 (95% CI, 0.19, 1.22; *I*^2^ = 0%) (Fig. 14).

#### Neurological Adverse Events

**Fig. 15:**
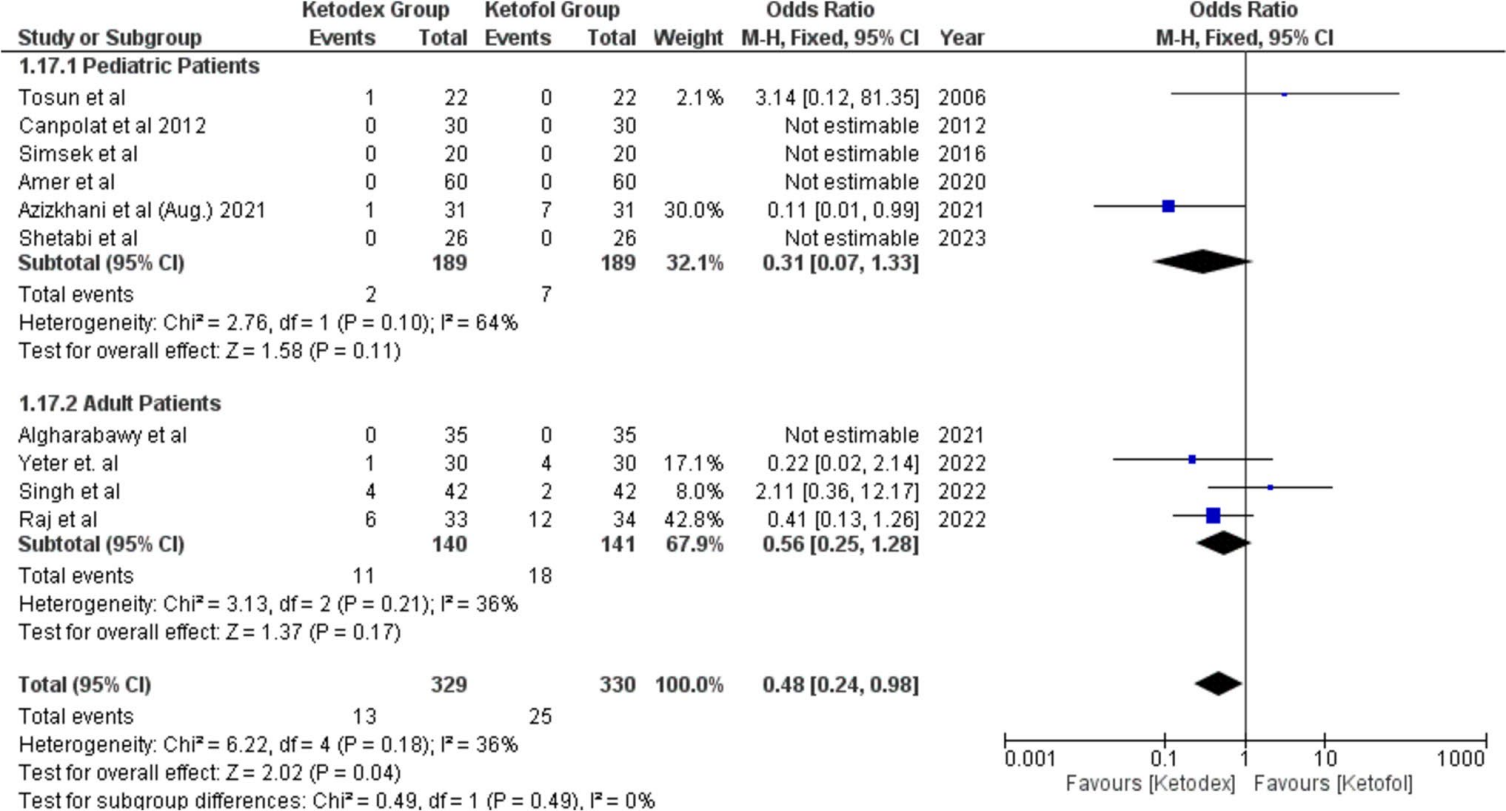
Forest plot of Recovery agitations outcome with age subgroups

Recovery agitations were reported in 29 patients of ten studies. The incidence in the ketofol group 7.6% was higher than in the ketadex group 4% with significant odds ratio of 0.48 (95% CI, 0.24, 0.98; *I*^2^ = 36%) (Fig. 15).

**Fig. 16:**
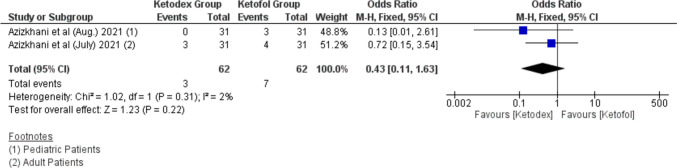
Forest plot of Hallucination outcome with age subgroups

Hallucination events were reported in only two studies in ten patients. Hallucination events appear to occur more in the ketofol group 11.3% while 4.8% in the ketadex group. However, there was insignificant odds ratio between both combinations 0.43 (95% CI, 0.11, 1.63; *I*^2^ = 2%). Both studies performed painful procedures in the emergency department (Fig. 16).

## Discussion

Although many agents were developed for procedural sedation, no magic bullet is available yet. This has been attributed to a lack of meeting the optimal sedation requirements such as rapid onset and offset, reversibility, and safe pharmacokinetic profile for various populations. Furthermore, the commonly used agents such as ketamine, dexmedetomidine, and propofol have their disadvantages as deleterious hemodynamic effects, compromising the airway reflexes, or poor pain control when used solely [33, 34]. Therefore, mounting research has been conducted to investigate the efficacy of their combination (ketadex and ketofol) to achieve the desired effect. Here, we gathered more comprehensive data comparing both combinations to help the anesthesiologists around the world make decisions on these agents in their daily practice.

### Pain Score

It has been established that both ketamine and dexmedetomidine provide analgesic effects in addition to providing sedation [35, 36]. The interesting fact here is that they work on different receptors in the pain pathway which advocate consideration of implementation in management of perioperative pain. Dexmedetomidine exerts analgesic effects via peripheral and central actions in the locus ceruleus and in the dorsal horn of the spinal cord, while ketamine has an agonist action on opioid receptors and antagonist effect on N-methyl-D-aspartate (NMDA) scattered throughout the central nervous system [37, 38]. In this regard, a systematic review and meta-analysis have demonstrated that propofol has no analgesic effect either in humans or animals [39]. However, many studies have demonstrated that prevention of pain is associated with propofol injection by combination with many other agents [40].

The effects on pain scores were evaluated in the present investigation because of its impact on patient satisfaction and composite outcome procedures. Further, the only previously conducted meta-analysis in the literature comparing the two combinations was in the pediatric population and did not investigate analgesic efficacy [10•].

In our meta-analysis, it was statistically significant (*P* = 0.0002) that both adult and pediatric populations experienced less pain in the ketadex group than in the ketofol group. Moreover, the mean difference was lower in adults than in pediatrics advocating their efficacy in controlling pain in adults more than the pediatric population. We acknowledge that there was moderate statistical heterogeneity in the pediatric group (*I*^2^ = 61%) related to the small number of conducted studies (two studies only) and their sample size in addition to the varied procedures being performed.

Therefore, ketadex should be considered in procedures with higher levels of postoperative pain.

### Hemodynamic Stability

Our analysis of the pooled data showed no difference between the two combinations regarding the incidence of hypertension and hypotension. This is basically explained by the balanced pharmacodynamics between ketamine (e.g., transient increase of the blood pressure related to sympathetic activity) and both propofol and dexmedetomidine (e.g., drop of blood pressure by decreasing the systemic vascular resistance and sympatholytic effect) [41]. Yang et al. reported the same findings in their meta-analysis comparing both combinations in the children [10•].

While the incidence of bradycardia was higher in the ketadex group, the present investigation revealed that tachycardia was higher in the ketofol group. However, both cardiovascular events were statistically insignificant. It is well established that ketamine increases heart rate while dexmedetomidine causes noticeable bradycardia by mediated or modulated effects at the alpha2-receptor. However, the meta-analysis performed by Yao et al. reported less bradycardia with addition of ketamine to dexmedetomidine rather than dexmedetomidine alone, advocating the combination over the single agent [42]. Propofol has an inhibitory effect on cardiac sodium, calcium, and potassium channels provoking bradycardia, yet Bradley et al. showed a slight increase of tachycardia when propofol was combined with ketamine in their meta-analysis [43•].

### Respiratory Adverse Effects

Airway compromise is a significant concern while sedating patients. This necessitates close monitoring of the respiratory function with pulse oximetry and quantitative end-tidal C02, along with attendance of an anesthesia provider, which typically increases financial costs for these procedures and selection of appropriate sedatives, especially for high-risk patients with difficult anatomical and co-morbidities [44].

In the present meta-analysis, subjects with ketadex (e.g., especially the pediatric population as a subgroup) experienced less hypoxia through different procedures than comparative subjects with ketofol. The incidence was statistically significant with an odds ratio of 0.49 and *P* value of 0.001.

Further, bradypnea and airway obstruction had the same low incidence in the ketadex group; however, both adverse outcomes were not statistically significant.

The same results were reported in the pediatric meta-analysis, which stated that ketadex was safer than ketofol with regard to respiratory adverse events [10•]. However, in the pediatric meta-analysis, the authors combined whole adverse events in one forest plot, while we differentiate respiratory complications into hypoxia or bradypnea.

The safety of ketadex had been linked to the fact that both ketamine and dexmedetomidine can preserve airway patency and pharyngeal musculature tone, while propofol possesses dose-dependent respiratory depression especially with boluses [45].

### Gastrointestinal Adverse Events

Post-operative nausea and vomiting are among the criteria which assess the readiness for discharge after procedural sedation [46]. Our meta-analysis showed a higher incidence of nausea and vomiting in subjects with ketadex than ketofol related most likely to the well-established antiemetic role of propofol [47].

However, in the pediatric subgroup, the incidence was statistically insignificant between the two groups; the same result was found by Yang et al. in their pediatric meta-analysis [10•].

In both the adult and pediatric population, the overall difference of the incidence of salivation which is provoked by ketamine (a common agent between the two groups) was statistically insignificant [48].

### Recovery

Prolonged recovery is a troublesome challenge for anesthesiologists with an impact on composite outcome involving hospital stay, especially in sedation procedures which are considered as day case surgery. One of the crucial risk factors of delayed recovery is the agent used for anesthesia and dosage. Therefore, factors have been studied in recent years to better control reversible elements including sedation technique, including choosing agents with shorter elimination half-time without residual effects and consideration of the role of potentiation and/or synergistic effects of medications [49]. Adding either dexmedetomidine or propofol appears to reduce both the incidence and severity of ketamine-induced recovery agitation in procedural sedation [27].

The recovery time in the present investigation was longer in the ketadex group than in the ketofol group which was demonstrated by Yang et al., who explained this by the properties of dexmedetomidine, including relative longer half-life in comparison to propofol [10•]. While Yang et al. found that recovery agitation was low in both groups, our investigation showed that the recovery agitation was greater in the ketofol group rather than in the ketadex group with statistical significance. Additionally, we evaluated hallucination and agitation incidence in two studies as separate outcomes and found greater agitation with ketofol, yet the difference was not statistically significant. In this regard, a meta-analysis was conducted in 2020 evaluating the role of dexmedetomidine in the prevention of emergence agitation which concluded that dexmedetomidine was an excellent choice to prevent emergence agitation [50]. Furthermore, a single bolus dexmedetomidine was more effective than a single bolus of propofol in treating the emergence delirium during the early postanesthetic stage [51]. It was not surprising that dexmedetomidine with its combined analgesic, sedative, and sympatholytic effect was proved to be superior to propofol on emergence delirium when compared with Huang et al. in 2022 [52].

### Physician Satisfaction

Since physician satisfaction is multifactorial, depending on many factors related to the physician, patient, and procedures, it should be noted that the clinician’s opinion was positive for both combinations with their satisfaction in our meta-analysis higher in the ketofol group in adult with statistical significance. However, the clinician satisfaction was insignificantly better with ketofol in the pediatric group. This might be related to recovery time and emergence vomiting, which were both higher in the ketadex group.

### Strengths and Limitations

To the best of our knowledge, this is the first meta-analysis to assess the safety and efficacy of ketadex versus ketofol based on all published RCTs in peer-reviewed journals until August 1, 2023. Additionally, we performed subgroup analysis according to age and procedure. However, the heterogeneity in the efficacy outcomes limits our meta-analysis. The heterogeneity may be related to different providers or age or dosage or other factors. Thus, we tried to overcome these limitations by evaluation of subgrouping analysis. Additionally, the limited number of published trials in certain subgroups makes our evidence and conclusions limited on some outcomes.

## Conclusion

Adding propofol and dexmedetomidine to ketamine in procedural sedation showed superior efficacy when compared to ketamine alone. Both facilitated the procedure, balanced the hemodynamic effects, and prevented the emergence delirium. However, in our meta-analysis, the pain score was obviously lower in subjects with ketadex than that receiving ketofol which no previous meta-analysis has demonstrated. Therefore, we advocate using ketadex in painful procedures or patients with higher pain threshold. Furthermore, ketadex was associated with lower hypoxic events and airway obstruction, which suggest consideration to select it in patients whose airway is at risk. Finally, ketadex was an attractive choice in patients who are at high risk for emergence delirium because of the established role of dexmedetomidine in preventing emergence agitation. However, ketofol had increased clinician satisfaction which might be attributed to shorter recovery time and lower incidence of nausea and vomiting. In patients with well-established risk factors for PONV, ketofol may be preferred related to the antiemetic effect of propofol. More studies are warranted to clarify best practice strategies in the future.

### Supplementary Information

Below is the link to the electronic supplementary material.

Supplementary file1 (DOCX 266 KB)

## Data Availability

No datasets were generated or analysed during the current study.
