# Supplementary material for: Efficacy and Safety of Ketamine-Dexmedetomidine Versus Ketamine-Propofol Combination for Periprocedural Sedation: A Systematic Review and Meta-analysis
Source: Curr Pain Headache Rep. 2024 Jan 12;28(4):211–27. doi: 10.1007/s11916-023-01208-0 (PMC10940385; doi:10.1007/s11916-023-01208-0)
Supplement: Supplementary file 1 — Supplementary file1 (DOCX 266 KB) [file 11916_2023_1208_MOESM1_ESM.docx]

**Efficacy and safety of ketamine-dexmedetomidine versus ketamine-propofol combination for periprocedural sedation: A systematic review and Meta-Analysis.**

**Running title**

Ketadex vs Ketofol

**Authors**

Ahmed Saad Elsaeidy ^1^, Aya Hisham Moussa Ahmad ^2^, Neveen A. Kohaf ^3^, Aya Aboutaleb ^4^, Danisha Kumar ^5^, Khaled Saad Elsaeidy ^6^, Ola saeed Mohamed ^7^, Alan D. Kaye ^8^, Islam Mohammad Shehata ^2*^

**Affiliations**

1- Faculty of Medicine, Benha University, Benha, Egypt.

[Ahmed.Saad.Elsaeidy@gmail.com](mailto:Ahmed.Saad.Elsaeidy@gmail.com)

<https://orcid.org/0000-0002-1643-9750>

2- Lecturer of Anesthesia department, Faculty of Medicine, Ain Shams University,

Cairo, Egypt.

3- Lecturer of Clinical Pharmacy, Faculty of Pharmacy (Girls), Al-Azhar University, Cairo, Egypt

4- Faculty of Medicine, Zagazig University, Zagazig, Egypt.

5- Dow Medical College, Dow University of Health Sciences, Karachi, Pakistan.

6- Faculty of medicine, Mansoura University, Mansoura, Egypt.

[khaled.saad.elsaeidy@gmail.com](mailto:khaled.saad.elsaeidy@gmail.com)

<https://orcid.org/my-orcid?orcid=0000-0002-3221-6338>

7- Lecturer of critical care medicine, Menofia University, Menofia Governorate, Egypt.

8- Dr. Alan David Kaye, MD, PhD, DABA, DABPM, DABIPP, CSSM, FASA

Editor-in Chief, Pain Physician, Current Pain and Headache Reports Pain Section, and Scientific American Pain Section, ASIPP and ABIPP Board of Directors; Immediate Former Vice-Chancellor of Academic Affairs, Chief Academic Officer, and Provost; Interventional Pain Fellowship Director, Vice Chairman of Research, and Tenured Professor of Anesthesiology; Professor, Pharmacology, Toxicology, and Neurosciences, LSU School of Medicine, 1501 Kings Hwy, Shreveport, LA 71103, Professor of Anesthesiology and Pharmacology, LSU School of Medicine, New Orleans, LA, Professor of Anesthesiology and Pharmacology, Tulane School of Medicine, New Orleans, LA. alan.kaye@lsuhs.edu, alankaye44@hotmail.com

* **Corresponding author**

Islam Mohammad Shehata

Lecturer of Anesthesia department, Faculty of Medicine, Ain Shams University, Cairo, Egypt.

[islam.shehata@med.asu.edu.eg](mailto:islam.shehata@med.asu.edu.eg)

<https://orcid.org/0000-0003-3663-3056>

**Index:**

[Table S 1. Databases search strategies and results. 3](#_Toc152623729)

[Figure S 1. Forest plot of Recovery time outcome with procedure subgroups. 4](#_Toc152623707)

[Figure S 2. Forest plot of Recovery time outcome with recovery score subgroups. 5](#_Toc152623708)

[Figure S 3. Forest plot of Pain score outcome with procedure subgroups. 6](#_Toc152623709)

[Figure S 4. Forest plot of Physician satisfaction outcome with age subgroups after the removal of Amer et al. 7](#_Toc152623710)

[Figure S 5. Forest plot of Bradycardia outcome with procedure subgroups. 8](#_Toc152623711)

[Figure S 6. Forest plot of Tachycardia outcome. 9](#_Toc152623712)

[Figure S 7. Forest plot of Hypoxia outcome with procedure subgroups. 10](#_Toc152623713)

[Figure S 8. Forest plot of Post-operative Nausea and/or vomiting outcome with procedure subgroups. 11](#_Toc152623714)

| Database | strategy | results |
| --- | --- | --- |
| Pubmed 7jun 2023 | ((((Precedex[Title/Abstract]) OR (Dexmedetomidine[Title/Abstract])) AND ((((((((Disoprofol[Title/Abstract]) OR (Diprivan[Title/Abstract])) OR (Disoprivan[Title/Abstract])) OR (Fresofol[Title/Abstract])) OR (Ivofol[Title/Abstract])) OR (Propofol[Title/Abstract])) OR (Recofol[Title/Abstract])) OR (Aquafol[Title/Abstract]))) AND (((((((Ketalar[Title/Abstract]) OR (Ketaset[Title/Abstract])) OR (Ketanest[Title/Abstract])) OR (Calipsol[Title/Abstract])) OR (Kalipsol[Title/Abstract])) OR (Calypsol[Title/Abstract])) OR (Ketamine[Title/Abstract]))) OR ((Ketofol[Title/Abstract]) AND (Ketodex[Title/Abstract])) | 254 |
| Cochrane | ((((((((((Disoprofol) OR (Diprivan)) OR (Disoprivan)) OR (Fresofol)) OR (Ivofol)) OR (Propofol)) OR (Recofol)) OR (Aquafol)) AND ((Dexmedetomidine) OR (Precedex))) AND (((((((Ketalar) OR (Ketaset)) OR (Ketanest)) OR (Calipsol)) OR (Kalipsol)) OR (Calypsol)) OR (Ketamine))) OR ((Ketodex) AND (Ketofol)) in Title Abstract Keyword | 213 Trials |
| Scopus | (( TITLE-ABS ( diprivan )  OR  TITLE-ABS ( disoprofol )  OR  TITLE-ABS ( disoprivan )  OR  TITLE-ABS ( fresofol )  OR  TITLE-ABS ( ivofol )  OR  TITLE-ABS ( propofol )  OR  TITLE-ABS ( recofol )  OR  TITLE-ABS ( aquafol ) )  AND  ( TITLE-ABS ( precedex )  OR  TITLE-ABS ( dexmedetomidine ) )  AND  ( TITLE-ABS ( ketalar )  OR  TITLE-ABS ( ketaset )  OR  TITLE-ABS ( ketanest )  OR  TITLE-ABS ( calipsol )  OR  TITLE-ABS ( kalipsol )  OR  TITLE-ABS ( calypsol )  OR  TITLE-ABS ( ketamine ) ) ) OR  ( TITLE-ABS ( ketodex )  AND  TITLE-ABS ( ketofol ) ) | 298 |
| WOS | 8- #7 OR #6  7- #1 AND #2 AND #3  6- #4 AND #5  5- TS=(Ketofol)  4- TS=(Ketodex)  3- TS=(((((((Ketalar) OR (Ketaset)) OR (Ketanest)) OR (Calipsol)) OR (Kalipsol)) OR (Calypsol)) OR (Ketamine))  2- TS=((Dexmedetomidine) OR (Precedex))  1- TS=((((((((Disoprofol) OR (Diprivan)) OR (Disoprivan)) OR (Fresofol)) OR (Ivofol)) OR (Propofol)) OR (Recofol)) OR (Aquafol)) | 516 |

Table S 1. Databases search strategies and results.


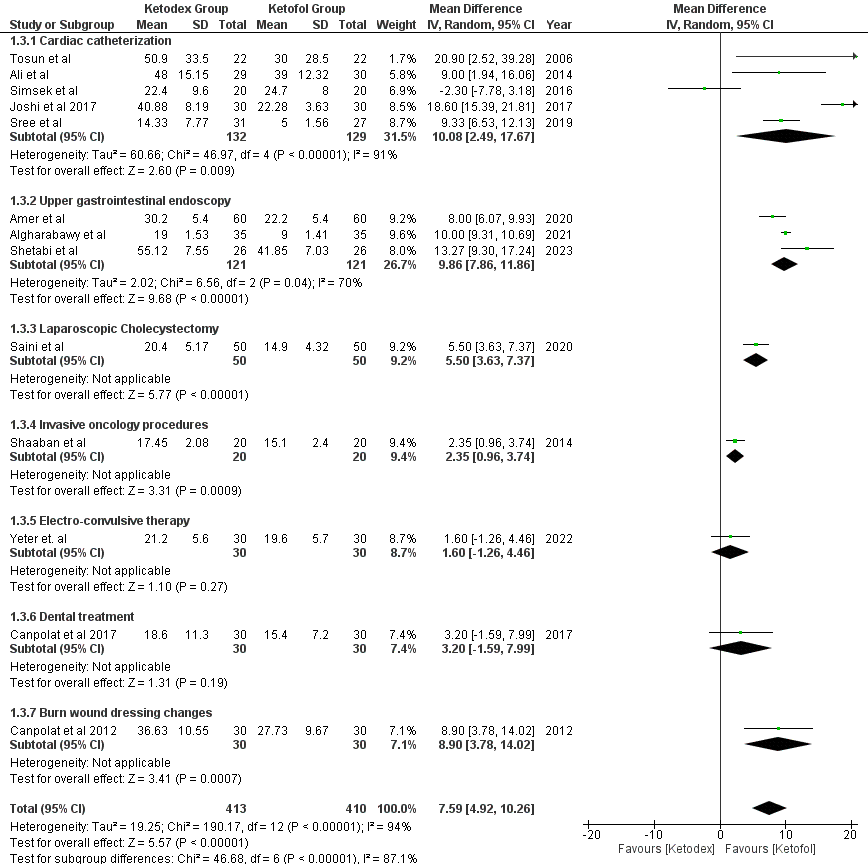


Figure S 1. Forest plot of Recovery time outcome with procedure subgroups.


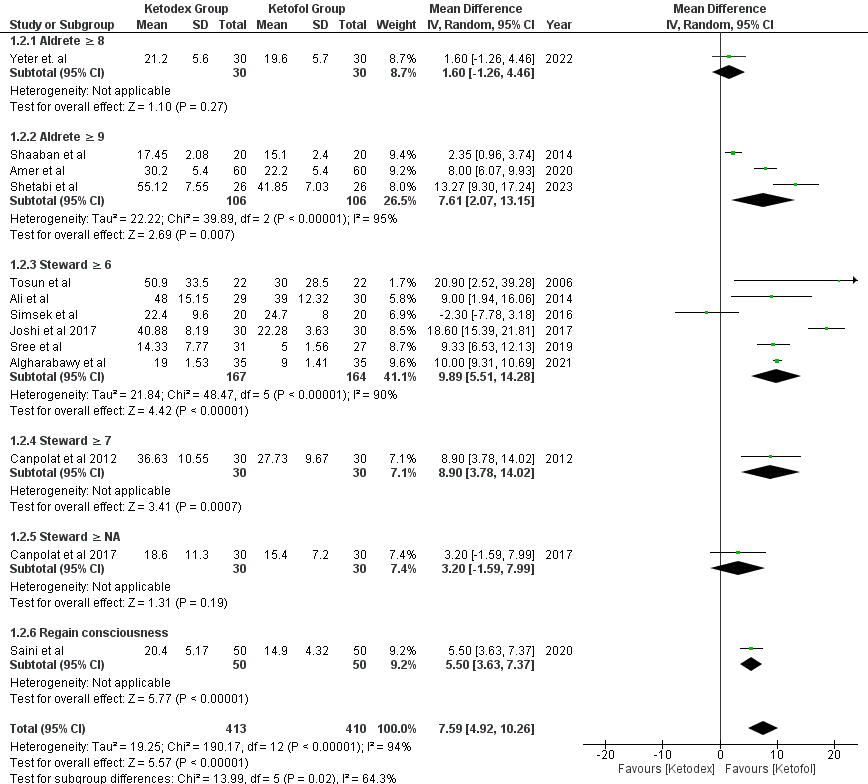


Figure S 2. Forest plot of Recovery time outcome with recovery score subgroups.


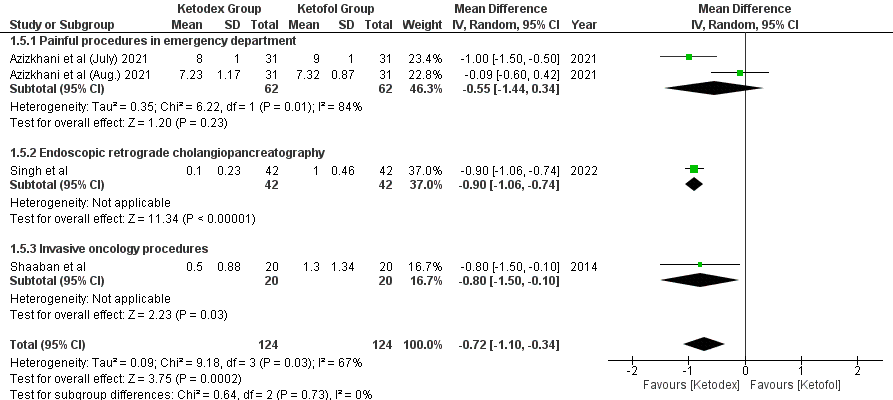


Figure S 3. Forest plot of Pain score outcome with procedure subgroups.


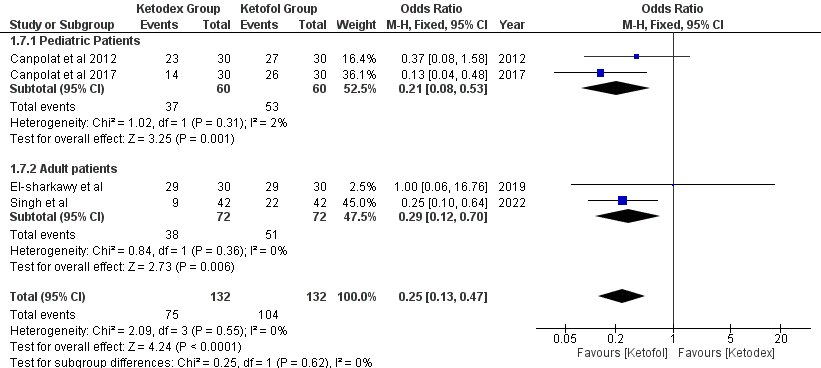


Figure S 4. Forest plot of Physician satisfaction outcome with age subgroups after the removal of Amer et al.


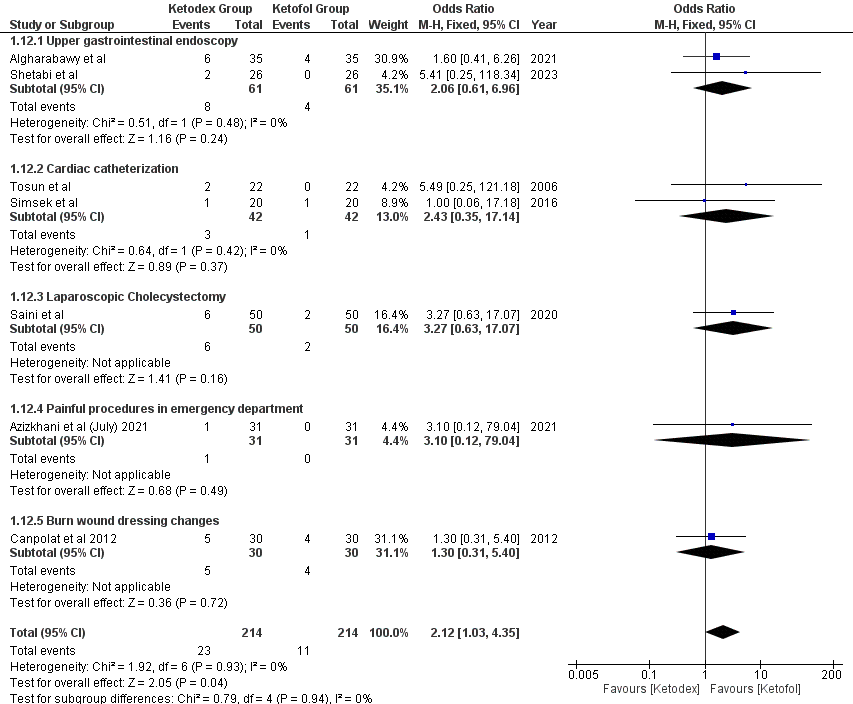


Figure S 5. Forest plot of Bradycardia outcome with procedure subgroups.


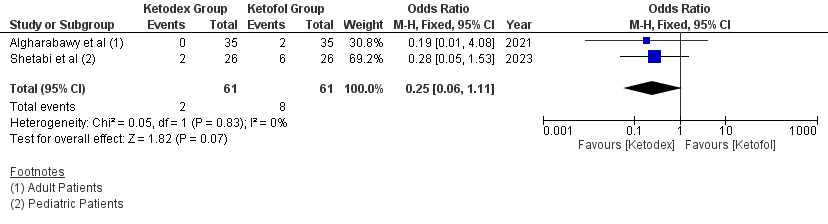


Figure S 6. Forest plot of Tachycardia outcome.


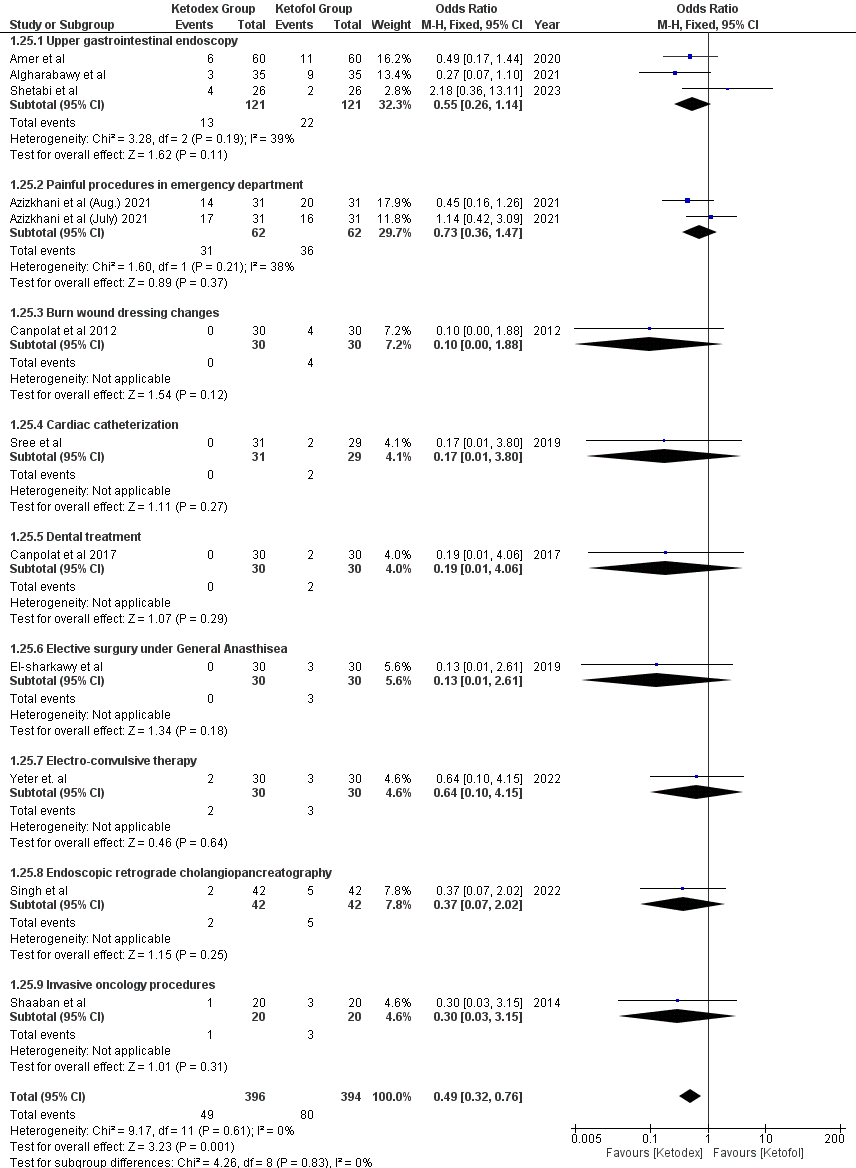


Figure S 7. Forest plot of Hypoxia outcome with procedure subgroups.


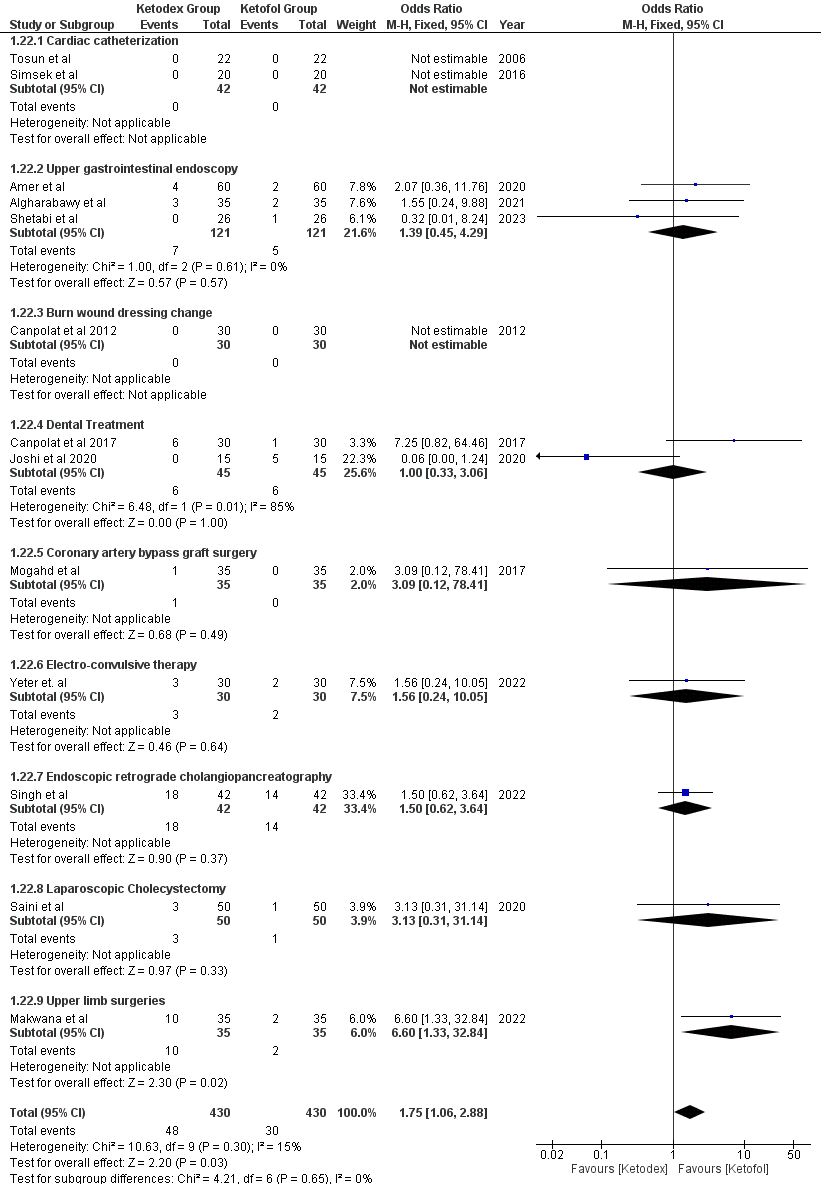


Figure S 8. Forest plot of Post-operative Nausea and/or vomiting outcome with procedure subgroups.
